# Visual Disengagement: Genetic Architecture and Relation to Autistic Traits in the General Population

**DOI:** 10.1007/s10803-019-03974-6

**Published:** 2019-03-11

**Authors:** Monica Siqueiros Sanchez, Erik Pettersson, Daniel P. Kennedy, Sven Bölte, Paul Lichtenstein, Brian M. D’Onofrio, Terje Falck-Ytter

**Affiliations:** 1 Center of Neurodevelopmental Disorders (KIND), Division of Neuropsychiatry, Department of Women’s and Children’s Health, Karolinska Institutet, 113 30 Stockholm, Sweden; 2 Center of Psychiatry Research, Stockholm County Council, 113 64 Stockholm, Sweden; 3 Department of Medical Epidemiology and Biostatistics, Karolinska Institutet, 171 65 Stockholm, Sweden; 4 Department of Psychological and Brain Sciences, Indiana University, Bloomington, IN 47405 USA; 5 Child and Adolescent Psychiatry, Stockholm County Council, 113 30 Stockholm, Sweden; 6 Department of Psychology, Uppsala University, 751 42 Uppsala, Sweden; 7 Swedish Collegium for Advanced Study (SCAS), Uppsala, 752 38 Sweden

**Keywords:** Visual disengagement, Gap-overlap task, Behavior genetics, Autistic traits, Autism spectrum disorders

## Abstract

Visual disengagement has been hypothesized as an endophenotype for autism. In this study we used twin modelling to assess the role of genetics in basic measures of visual disengagement, and tested their putative association to autistic traits in the general population. We used the Gap Overlap task in a sample of 492 twins. Results showed that most of the covariance among eye movement latencies across conditions was shared and primarily genetic. Further, there were unique genetic contributions to the Gap condition, but not to the Overlap condition—i.e. the one theorized to capture visual disengagement. We found no phenotypic association between autistic traits and disengagement, thus not supporting the hypothesis of visual disengagement as an endophenotype for autistic traits.

**Electronic supplementary material:**

The online version of this article (10.1007/s10803-019-03974-6) contains supplementary material, which is available to authorized users.

## Introduction

Attention is a selection process that helps us focus on certain aspects of the world and, in consequence, filter others out (Raz and Buhle 2006; Desimone and Duncan 1995; Posner and Petersen 1990). How we allocate our attention impacts the way we experience the world and how we interact with it. There is substantial variability between individuals in tasks of visual attention (Fischer et al. 1993), and according to one hypothesis, atypicalities in attentional processes could be important for our understanding of autism (Keehn et al. 2013), a neurodevelopmental condition defined by problems with social communication and restricted and repetitive behaviors and interests. The idea is that early emerging dysfunctions in the processes of visual attentional allocation (e.g. moving gaze effortlessly from one location to another) could have cascading effects on individuals’ development by disturbing the building of foundational blocks for sociocommunicative skills (e.g. perception and joint attention which are often impaired in autism). Being able to flexibly and quickly move ones gaze between stimuli could have consequences for one’s ability to regulate one’s arousal (e.g. failing to regulate eye contact during face to face communication) (Rothbart et al. 2008; Bryson et al. 2018). Although gaze location is not always a reliable indicator of someone’s focus of attention in a given moment, typically a close relation exists between the two, and the neural networks for eye movements and attentional processes are largely overlapping (Hutton 2008; Corbetta et al. 1998).

In line with the idea that attentional atypicalities could be important for our understanding of autism, there is some evidence suggesting that problems with disengagement of attention are present in infancy in individuals who are later diagnosed with the disorder (Elsabbagh et al. 2013; Elison et al. 2013). Specifically, delayed visual disengagement (longer latencies) was reported in 7 month-old infants (Elison et al. 2013) and 14 month-old infants (Elsabbagh et al. 2013) who later went on to display autism symptoms and receive a diagnosis. In contrast to the studies of infants at risk for autism, case-control studies of disengagement of attention in children and adolescents with autism have yielded mixed results. Comparing groups with autism with controls, some studies have found slower (Landry and Bryson 2004; Goldberg et al. 2002; Wainwright-Sharp and Bryson 1993; Kleberg et al. 2017), faster (van der Geest et al. 2001), and no differences between (Fischer et al. 2014, 2016; Mosconi et al. 2009) disengagement times (latencies). Paradigm differences seem to contribute to some of these discrepancies, for reviews and discussions see Sacrey et al. (2014) and Johnson et al. (2016). Studies assessing visual disengagement have also reported decreased accuracy (undershooting) of the saccades of individuals with autism (Schmitt et al. 2014; Luna et al. 2007; Johnson et al. 2012).

It is well established that genetic factors play an important role in autism. Research on the contribution of genetics and environments to attentional measures, however, is scarce. Two recent twin studies found genetic influences on where one looks within highly complex naturalistic stimuli during childhood (Constantino et al. 2017; Kennedy et al. 2017). In contrast, a small study looking at visual orienting (in adults) found no evidence of it being heritable (Fan et al. 2001). Because of these large discrepancies in heritability estimates, there is a need to clarify the role of genes for variability in lower level (non-social) attentional functions, as well as the association between these functions and neurodevelopmental conditions such as autism.

Visual disengagement has been widely studied using the Gap-Overlap paradigm (Johnson et al. 2016). This task is composed of three different experimental conditions which measure, in units of time, the ease with which the participant is able to disengage from a visual stimulus to orient to a new one across three increasing levels of visual competition (from none to high competition: gap < baseline < overlap). The conditions elicit different behavioral responses and are presumed to capture distinct attention related processes (Fischer and Breitmeyer 1987) with different neural correlates reported for the Gap and the Overlap experimental conditions (Csibra et al. 1997; Gómez et al. 1996).

In this study, we used eye-tracking in a twin study to understand the contribution of genes and environments to visual attention and to the putative link between visual attention and autistic traits in the general population. The approach allowed us to test the hypothesis that visual disengagement is an endophenotype (heritable underlying trait) for variability in social communication and non-social obsessive behaviors (strong interests, insistence on sameness) in the general population and at the extreme: autistic-like symptoms.

The study focuses on autistic traits rather than autism diagnoses, but it is important to note that several lines of evidence support a dimensional view of autism, and hence a relation, e.g. in terms of etiology, between autistic traits and autism diagnoses. For example, family studies of individuals with autism report elevated levels of autistic traits (Constantino et al. 2006), and highly similar heritability estimates and shared etiologies for the extreme (up to 1%) and subthreshold variations of autistic traits (Lundstrom et al. 2012; Robinson et al. 2011). Studies also show that part of the genetic etiology for autism comes from common polygenic variation, with most genetic risk factors for autism being found in the general population (Gaugler et al. 2014; Klei et al. 2012). Furthermore, recent evidence suggests that variation in sociocommunicative abilities in the general population is influenced by genetic factors that also influence autism risk (Robinson et al. 2016). Polygenic risk score studies render partial support but results are mixed (St Pourcain et al. 2018; Martin et al. 2017). Therefore, studying autistic traits in the general population can be relevant for our understanding of the clinical manifestations of the condition.

As visual disengagement atypicalities in autism have been linked specifically to performance in the Overlap condition of the task (where demands for disengagement are thought to be highest due to the competition between the two visual stimuli), we expected (1) that there would be unique genetic influences on eye movement latencies (visual disengagement) in this specific condition, and (2) that longer latencies of eye movements in this condition would be associated with higher levels of autistic traits. We also expected that saccade accuracy would be negatively correlated with autistic traits, based on the previous findings mentioned above (Schmitt et al. 2014; Luna et al. 2007; Johnson et al. 2012), and that saccade accuracy would have a substantial genetic component.

## Methods

### Participants

Children aged 9–14 years recruited from the longitudinal Child and Adolescent Twin Study in Sweden (CATSS) (Anckarsater et al. 2011). The CATSS study is a nation-wide population longitudinal twin study with a 70% inclusion rate, aiming to understand how genes and environment influence behavior and health in childhood and adolescence. In CATSS, information on mental and physical health about the twins is collected through a telephone interview with the twins’ parents using a set of standardized questions. Twins already participating in CATSS (CATSS 9 year assessment) were invited to take part in the present study which would involve an additional set of assessments (parental-report measures on ASD traits and ADHD behaviors, a background questionnaire, and a cognitive assessment) and an eye-tracking task (details on the measures and the procedure can be found in the following paragraphs). To be included in the study, the participants’ parents had to be fluent in Swedish. Opposite-sex twins, individuals with severe uncorrected hearing or vision impairment, known presence of a genetic syndrome, presence of known significant medical condition likely to affect brain development or the child’s ability to participate in the study (e.g. Cerebral Palsy, Down’s syndrome, cystic fibrosis), and twins with missing values on their co-twin were excluded. Zygosity was determined by molecular genetic analysis. In a few cases where DNA samples were not available, a highly accurate five item questionnaire on twin similarity was used to determine zygosity (Anckarsäter et al. 2011). Eye-tracking data was collected from 723 children but after quality control procedures (see section on “Analysis of Eye-Tracking Data” for more details) and selecting those with complete data, only 492 twins could be included in the twin modelling analyses (reflecting also that inclusion required valid data from both twins in the pair).

**Fig. 1:**
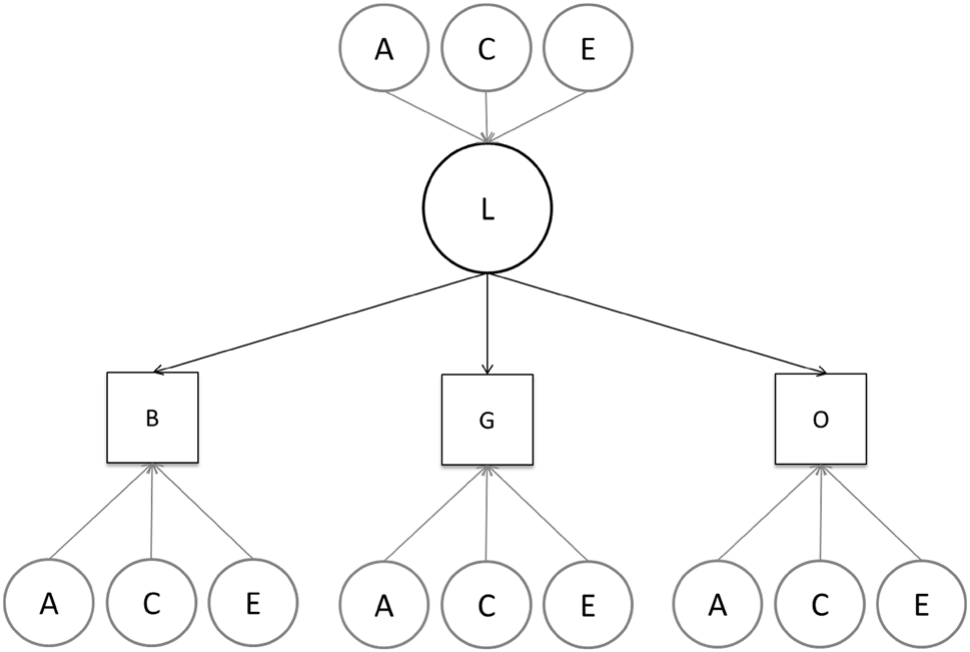
A common pathway model produced the best fit for both leaving latency and saccadic amplitude. In small circles, A = Genes, C = Shared environment, E = Non-shared environment. Large circle, L = Common latent factor. Squares, B = Baseline, G = Gap, O = Overlap

The final sample consisted of 120 monozygotic (MZ; 63.3% females) pairs and 126 dizygotic (DZ; 60.3% females) pairs, with a mean age of 11.25 (*SD* = 1.28) years, with a majority of the twins having highly educated parents (44% of fathers and 52% of mothers with ≥ 3 years of university/college studies or higher). The present study sample is largely representative of the larger CATSS sample on terms of sex, but with overall maximum parental education level being slightly higher than in the CATSS sample (please refer to figure 1 in the online resource for a comparison of the two samples). In this sample, 16 individuals (3.25%) had received a psychiatric disorder diagnosis of some kind and 4 (0.8%) of ASD (by a healthcare related professional/service as reported by the parents) by the time of the assessment in this study. Further participant characteristics can be found in Tables 1 and 2.

**Table 1:** Descriptive statistics for demographic and phenotypic variables (raw values)

	Age	Autistic traits (SRS)	IQ*
Males (*n* = 188)			
*M* (SD)	11.26 (1.30)	24.38 (18.41)	9.5 (1.55)
Range (min, max)	4.62 (9.27, 13.88)	127 (2, 129)	10 (5.3, 15.3)
Skew	0.46	2.55	2
Kurtosis	− 0.76	9.22	0.8
Females (*n* = 304)			
*M* (SD)	11.24 (1.27)	20.25 (13.87)	9.94 (1.66)
Range (min, max)	4.92 (9.22, 14.14)	87 (1, 88)	10.3 (3.7, 14)
Skew	0.68	1.52	− 0.30
Kurtosis	− 0.51	3.30	0.31

*SRS* Social Responsiveness Scale (total score). * The IQ scale is the average of the standard score obtained from each subscale (n = 4). It typically has a mean of 10 and a sd of 2.5

**Table 2:** Frequencies of high autistic traits and clinical diagnosis (any and ASD)

Percentile	Raw score cutoff	Z-score cutoff	No. of individuals	No. of males (%)	SRS cutoff’s (no. of individuals)	No. of males (%)
Any dx	–	–	16	12 (75)	–	–
ASD dx	–	–	4	3 (75)	–	–
≥ 99%	84	3.62	4	3 (75)	85 (*n* = 4)	3 (75)
≥ 97.5%	61	2.81	11	7 (63.6)	75 (*n* = 10)	7 (70)
≥ 95%	52	1.98	23	12 (52.2)	–	–
ALL	–	–	492	188 (38.2)	–	–

Any dx: Any diagnosis present at test as reported by a parent via telephone interview. ASD dx: Any diagnosis of ASD present at test (all cases had an Autism diagnosis)

Written informed consent to participate in the study was obtained from the parents of all the twins. The study was approved by the local ethical committee in Stockholm, and was conducted in accordance with the 1961 Declaration of Helsinki.

### Procedure

The testing session consisted of one eye-tracking experimental battery (including the Gap-Overlap task and several other experiments not related to the current research questions; Kennedy et al. 2017) and a cognitive assessment, and took 60 min in total. For the eye-tracking task, the participant sat in front of the eye tracker and the screen displaying the stimuli at a distance of approximately 60 cm; the research assistant administering the experiment remained out of sight from the participant after giving verbal task instructions. For the cognitive assessment, a clinical psychologist (or a student of clinical psychology under supervision by TFY) administered four subscales of the Wechsler Intelligence Scale for Children IV (WISC-IV) (Wechsler 2003) in a separate room. While one twin performed the eye-tracking task, the other performed the cognitive task and the parent(s) completed electronic questionnaires about the twins. For more details on each measure please refer to the corresponding sections (eye-tracking and psychological and cognitive assessments).

### Visual Disengagement Task

The Gap-Overlap task was used to operationalize visual disengagement. This task includes three conditions: Gap, Baseline and Overlap (although some studies only use the conditions Gap and Overlap). Common to all conditions is that a central stimulus appears on a screen and is followed by a new stimulus that appears in the periphery, but they differ with regards to when the central stimulus disappears in relation to when the peripheral stimulus appears. In the “Gap condition” the central stimulus disappears before the peripheral stimulus appears. Hence, in this condition, there is no stimulus to disengage from, and the disappearance of the central object may function as a (spatially non-predictive) preparatory cue. In the “Baseline condition” the central stimulus disappears simultaneously as the peripheral stimulus appears; hence in this condition, no preparatory cues are given, but there is also nothing to disengage from at the moment the peripheral stimulus appears. Finally, in the “Overlap condition” the central stimulus remains displayed when the peripheral stimulus appears. The time it takes to execute a saccade (saccadic reaction time) in this condition is the longest; this is thought to reflect that they have to disengage their gaze (fixation) from the central stimulus in order to orient to the peripheral one (Fischer and Breitmeyer 1987). Different saccadic reaction times are obtained in the conditions with saccadic reaction times being of shorter duration in the Gap condition (150 ms) than those in the Overlap condition (250 ms).

In all stimuli, a central stimulus (CS) appeared on a gray background and was followed by a new stimulus that appeared on the periphery (PS). The CS consisted of a black cross and the PS of a yellow circle, both were 1.5° visual degrees wide. In all conditions, the CS appeared in the center of the screen and was followed by the PS. In the Gap condition, the CS disappeared 200 ms before the PS appeared. In the Baseline condition the CS disappeared when the PS appeared. In the Overlap condition the CS remained on the screen when the PS appeared. A total of 72 trials were presented in this task, with 24 trials per condition. The target appeared on the left side of the screen in half of the trials and to the right on the other half. There were two trial duration lengths, with half of the trials lasting a total of 4200 ms (long) and half a total of 3800 ms (short). The difference consisted of X (the time the fixation cross was displayed), which was 1600 in the long trials and 1200 in the short trials. Each participant saw a unique pseudorandom order (Side (left, right), Condition (Gap, Baseline, Overlap) and Duration (short, long)).

The main dependent variable was visual disengagement defined as the median average of saccadic reaction times in milliseconds (i.e., time taken to move away one’s gaze from the CS to the PS) from all conditions, and is also referred to as the leaving latency (see the “Analysis of Eye Tracking Data” section for more detail).

### Psychological and Cognitive Assessments

#### Autistic Traits

Parents completed the Social Responsiveness Scale (SRS) (Constantino and Gruber 2005), as a measure of autistic traits. The raw total score of the SRS was used in the analyses. The SRS is a much used parent-report instrument assessing autistic traits, and is sensitive to a continuum of social impairment severity thus also capturing subthreshold social deficits (Constantino et al. 2003). The SRS total score has high sensitivity and specificity for any autism spectrum disorder using a 75 as a cut-off point (0.85 and 0.75 respectively) and a 85 cut-off total score (0.70 and 0.90 respectively) (Constantino and Gruber 2009). The SRS total score correlates well with the Autism Diagnostic Interview-Revised (ADI-R) one of the gold-standard clinical measurements for ASD (Bölte et al. 2011; Constantino et al. 2003), indicating construct validity. In this 65-item parental rating scale, parents are instructed to rate how well each statement describes their child’s behavior during the past 6 months using a 4-point Likert scale. Items found in this scale address both social (e.g. “avoids eye-contact or displays unusual eye-contact”, “knows when he/she is too close to someone or invading someone’s space”) and non-social (e.g. “has repetitive, odd behaviors, such as hand flapping or rocking”, “thinks or talks about the same thing over and over again”) autistic-like behaviors.

#### Cognitive Assessment

The cognitive assessment consisted of a clinical psychologist administering four subscales of the WISC-IV to each twin individually. The WISC-IV is a valid and widely used assessment for intelligence ability, providing both an overall score of intelligence as well as functioning scores for its subscales (Wechsler 2003). In this study, standard scores from the vocabulary, digit span, coding, and matrix reasoning subscales were averaged (expected *µ* = 10, SD = 2.5) to provide an index of cognitive ability as a proxy for IQ. IQ is included as a covariate since it can be a potential confounder when assessing phenotypic correlations between autistic traits and performance on the Gap-Overlap task.

In addition, the study included parental ratings of various aspects of child behavior beyond the ones mentioned above. These measures were not part of this study and thus were not used in the current analysis.

### Eye-Tracking

Eye movements during the Gap-Overlap task (see above) were recorded using a Tobii T120 eye tracker at 120 Hz sampling rate. The stimuli were displayed as full-screen on a 23″ monitor with a 1024 × 1280 pixel resolution. A 9-point calibration image was used to determine the positions of the eyes before the task began. The task begun only after a successful calibration was achieved according to the experimenter (repeated if necessary). Glasses and contact lenses were allowed as long as vision was normal after this correction.

To quantify the (time) accuracy of the eye tracker and data recording software, prior to study start we performed a test of its performance during the Gap Overlap paradigm. We compared reported latencies with latencies computed from recordings from a high speed camera filming the eyes of the participants and the stimulus screen simultaneously (using a mirror located behind the child). The test was performed on three individuals, using a dedicated script embedded in the TimeStudio software (Nyström et al. 2016). Timing performance was excellent, with an average difference between the reported (eye tracker based) and observed (video based) latencies of 4.12 ms (sd = 19.37 ms).

### Analysis of Eye Tracking Data

Data were analyzed using custom scripts written in MATLAB (MathWorks; available upon reasonable request). Means, medians and standard deviations for saccadic reaction times were calculated for each participant in each condition. Three areas of interest (AOI) were defined for the analysis; one covering the fixation cross displayed in the middle of the screen (central AOI), and two peripheral AOIs covering the peripheral targets that appeared at both sides of the screen (left and right peripheral AOIs).

The primary dependent variable was “Leaving Latency”. In addition, we also calculated a dependent variable labelled “Arriving Latency” as well as “Saccade Amplitude”. The Leaving Latency was defined as the difference between the time the peripheral target appeared and the time the gaze first exited the central AOI. The Arriving Latency was defined as the difference between the time the peripheral target appeared and the time the gaze first entered the peripheral AOI. The Saccade Amplitude was chosen as a measure of saccadic accuracy. It was calculated for the first saccade executed after the peripheral target appeared. It was defined as the difference between the x-coordinate of the first fixation point outside the central AOI and the median x coordinate of gaze within the central AOI. This median was based on all the gaze data points registered inside the central AOI during the entire time interval that it was displayed in the current trial.

**Fig. 2:**
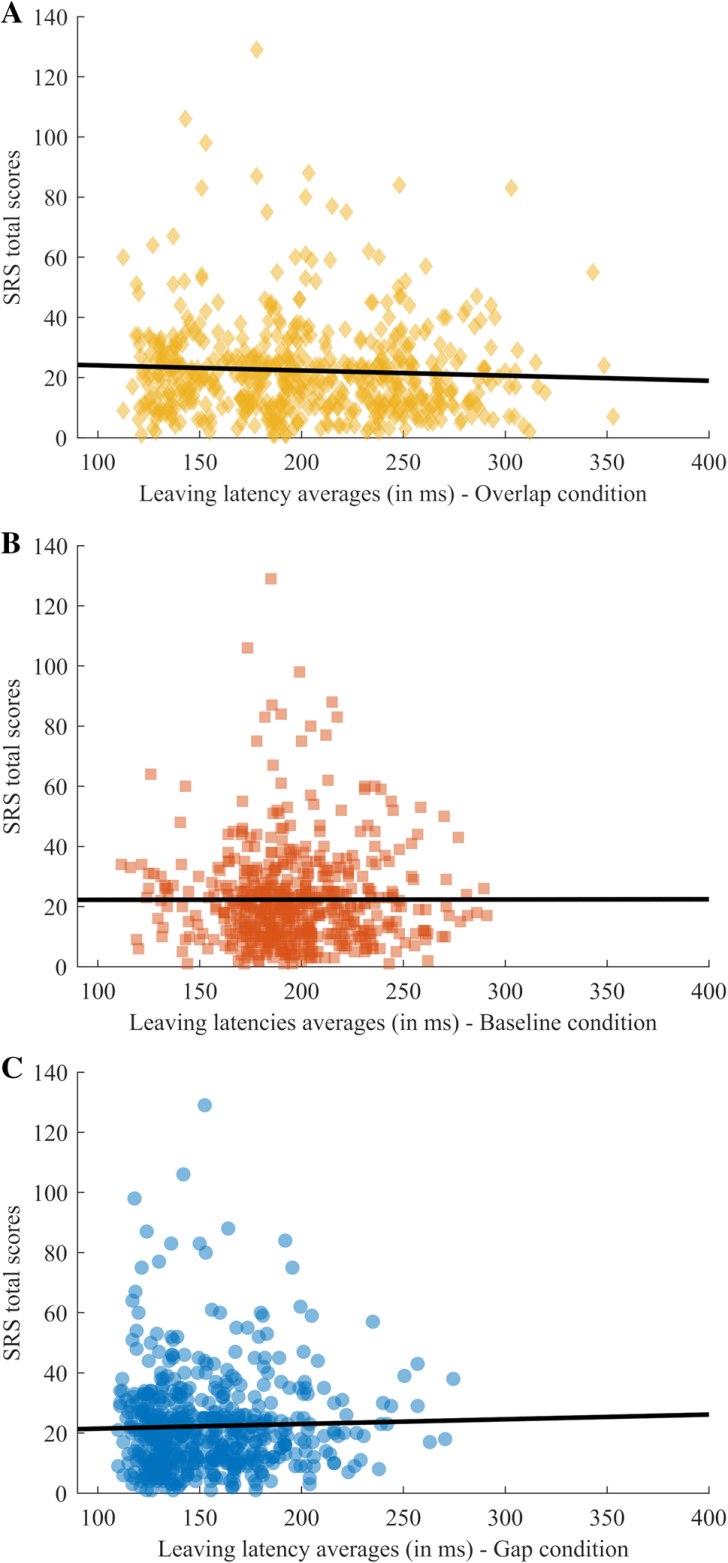
Leaving latency averages plotted against the SRS total scores for each condition with a fitted regression line (all P > .25). Marker color for each condition: Gap (**a**), Baseline (**b**), Overlap (**c**)

The three dependent variables were only calculated if a set of inclusion and inclusion criteria were met. Specifically, we required that (1) Gaze was within the central AOI for at least 50% of the time the fixation cross was displayed prior to peripheral target onset. This ensured that participants had looked at the fixation cross prior to the gaze shift and that trials with substantial data loss were excluded. (2) Valid gaze data was found inside the central AOI for at least 50% of the samples during the last 200 ms before the gaze first exited it. This ensured that the leaving latencies were not based on spurious data. (3) After peripheral target onset, gaze data was found within the peripheral AOI. This ensured that the gaze ultimately arrived at the peripheral AOI. (4) After peripheral target onset, no gaze points were recorded on the opposite side of the central AOI. This ensured that trials where participants first moved their gaze to the opposite side of the fixation cross were excluded. Finally, (5), we required that the first gaze data sample within the peripheral AOI was part of a fixation, defined as at least 50% of the gaze data during the subsequent 200 ms being within the peripheral AOI and no gaze data outside the peripheral AOI during this period. In addition, latency values were only included if they were above 60 ms to ensure it was not a predictive saccade. This threshold was supported by visual inspection of the data (see figure 2 of the online resource). Finally, if a participant had less than four valid trials per condition the participant (and his twin) was excluded from further analyses. This minimum represented a good balance between maximizing inclusion of pairs (complete pairs needed for twin analysis) and building each person’s measures on as many trials as possible, and it was selected after preprocessing of the data but before running the main analysis.

In the case of Saccade Amplitude, if criteria 1–5 were fulfilled and there were at least four valid trials per condition, this variable was calculated based on the first saccade that exited the central AOI. To determine if the first saccade landed between the central AOI and the peripheral AOI, we adopted a “moving AOI” approach (Flanagan and Johansson 2003), as a means of checking if the first data point located in this area was part of a fixation. If the gaze point was followed by at least 200 ms of data within a radius of 1.3° (i.e. similar size as the other, stationary, AOIs) we considered it being part of a fixation. If this was the case, this value was used to calculate Saccade Amplitude. If no data was detected between the two stationary AOIs, the first gaze point inside the peripheral AOI was used to calculate the Saccade Amplitude.

### Statistical Analysis

#### Preliminary Analyses on the Gap-Overlap Task

Before pursuing twin modelling analyses and phenotypic correlations between visual disengagement and autistic traits, correlations between conditions in the leaving and arriving latencies were calculated, and the expected effect of condition—a stepwise pattern (Gap < Baseline < Overlap)—in all variables was verified using a series of repeated measures ANOVAs. Correlations between all conditions for the leaving latency and for the arriving latency (Table 1 of the online resource) were moderate to high, and significant. In the Gap-Overlap task, saccadic reaction times (latencies) typically follow (descriptive and normality statistics for all variables on each condition and data distributions are presented in Table 2 and figures 3–5 of the online resource). In cases where the assumption sphericity was violated according to Mauchly’s sphericity test, we used the Huynh-Feldt estimates for sphericity. Results for the ANOVAs can be found in full in the online resource but in sum, a main effect of condition and in a stepwise pattern was found for both the Leaving latency (*F*(1.71, 988.83) = 584.76, *p* < .001; sphericity not assumed) and Saccade amplitude (*F*(2, 1154) = 192.349, *p* < .001). However, pairwise comparisons in the Arriving latency indicate that Baseline and Gap conditions were not significantly different from each other (*p* = .359). Additionally, in line with previous research (Fischer et al. 1997), the Gap condition was not only characterized by the shortest reaction times in the leaving latency, but also by the shortest saccadic amplitudes (figure 6 of the online resource). This pattern highlights that arriving latency is unlikely to be a pure measure of visual disengagement, rather a measure reflecting a series of processes including how early gaze moves away from the starting position, as well as planning and motor processes determining the spatial accuracy of the eye movements towards the next target. Due to this complexity, we did not pursue a twin modeling analysis of arriving latency, but used the leaving latency as our measure of timing of eye movements.

#### Twin Modelling

Analyses were conducted in Mplus version 7.31 (Muthén and Muthén 1998–2010). In the classic twin design, the genetic relatedness of twins (100% for MZ; 50% DZ) is correlated with the phenotypic similarity on a trait between pairs. If MZ twins are more similar on a trait than DZ twins, this implies that genetic effects influence the trait. If the similarity between MZ is roughly the same as the one between DZ, this implies that the shared environment influences the trait. Therefore, in addition to the estimation of additive genetic effects of the variance of one trait (A; heritability), the twin design allows to further partition environmental influences into shared (C; e.g. family environment, socio-economic status) and non-shared (E; e.g. illness at the day of testing) (Verweij et al. 2012). Univariate analyses were used to estimate the heritability of performance on each condition. Multivariate analyses were used to assess independence of the genetic and environmental influences on each condition. In multivariate analyses, cross-twin cross-trait correlations are used to partition the covariance between traits (in this case conditions) into genetic and environmental influences. Three types of models were fitted in order of decreasing complexity: the correlated factors model, the independent pathway model, and the common pathway model (Rijsdijk and Sham 2002). In the correlated factors model, which is the least constrained model, the sources of variance (genetic and environmental) are allowed to correlate between phenotypes, these correlations (r) can range from zero, no overlap, to 1, a complete overlap. In the independent pathway model, which is a more restricted model, genetic and environmental factors influence the response variables separately. In the common pathway, which is the most constrained model, it is assumed that a single latent phenotypic factor influences variations among the response variables, and this single latent variable is in turn influenced by genetic and environmental factors (Fig. 1). To assess the best fitting model to the data, we used the Bayesian Information Criteria (BIC) goodness of fit statistic. BIC identifies the most parsimonious model by weighing both how well the models fits the data, and how few parameters it uses. This index is designed this way because a model that uses more parameters always fits the data better, but such a model might not be the most parsimonious. Lower BIC values indicate a better fit of the model to the data (Raftery 1995). Intellectual ability, sex and age at testing time were regressed out of all of twin analyses.

## Results

### Twin Modelling Results

As noted in the preliminary analyses, only leaving latencies (not arriving latencies) and saccade amplitude were included in the twin modeling analyses.

#### Intra-Class Correlations

Intra-pair Pearson correlations were computed for the three conditions on all dependent variables (Table 3) and for autistic traits. Lower correlations in dizygotic twins compared to monozygotic twins in all three variables and conditions suggest genetic effects on performance on the task (e.g. saccadic reaction times). Correlations of autistic traits (SRS total score) were higher for MZ twins (*r* = 0.611; 95% CI 0.49, 0.72) than for DZ twins (*r* = 0.116; 95% CI − 0.06, 0.29), suggesting genetic effects on autistic traits.

**Table 3:** Intra-class Pearson correlations between MZ and DZ twins for arriving latencies and saccade amplitude on the three conditions

	Gap	Baseline	Overlap
Leaving latencies			
MZ	0.62 (0.50, 0.74)*	0.54 (0.40, 0.68)*	0.42 (0.26, 0.58)*
DZ	0.28 (0.12, 0.44)*	0.10 (− 0.08, 0.28)	0.15 (− 0.03, 0.33)
Saccade amplitude			
MZ	0.67 (0.57, 0.77)*	0.59 (0.47, 0.71)*	0.41 (0.25, 0.57)*
DZ	0.24 (0.08, 0.39)*	0.01 (− 0.17, 0.19)	0.16 (− 0.02, 0.34)

95% confidence intervals (CI) estimates are given in parentheses. *p < .05

#### Univariate Analyses

Analyses confirmed that both dependent variables (leaving latency; saccade amplitude) were influenced by genetic factors (Table 4). Overall, there were moderate genetic effects for the two variables in the three conditions (*h*^2^ between 0.38 and .66). There were no or non-significant contributions from shared environment. The rest of the variance was explained by non-shared environmental effects. Based on the autistic traits correlation of monozygotic being more than twice the magnitude of the autistic traits correlation in dizygotic twins, which suggests dominance effects, an ADE model was fitted. Autistic traits were primarily influenced by dominance genetic effects (D = 0.62; 95% CI 0.45, 0.79), while additive genetic effects (A = 0.14; 95% CI − 0.30, 0.58) and non-shared environment (E = 0.24; 95% CI − 0.17, 0.65) were non-significant.

**Table 4:** Univariate estimates of genetic and environmental contributions to the leaving latency and the saccade amplitude

	Genetic	Environment	
	*h*^2^	Shared c^2^	Non-shared e^2^
Leaving latency			
G	0.55 (0.33, 0.76)*	0.05 (− 0.13, 0.23)	0.40 (0.30, 0.50)*
B	0.48 (0.34, 0.62)*	0 (− 0.04, 0.04)	0.52 (0.38, 0.66)*
O	0.38 (0.22, 0.54)*	0.02 (− 0.06, 0.10)	0.61 (0.47, 0.75)*
Saccade amplitude			
G	0.68 (0.58, 0.78)*	0 (0, 0)	0.32 (0.22, 0.42)*
B	0.47 (0.33, 0.61)*	0 (0, 0)	0.53 (0.39, 0.67)*
O	0.46 (0.36, 0.56)*	0 (0, 0)	0.54 (0.44, 0.64)*

*G* Gap, *B* baseline, *O* Overlap. 95% confidence intervals (CI) estimates are given in parentheses. *p < .05

#### Multivariate Analyses

In these analyses, the BIC statistic, which was the goodness of fit statistic used in this study (lower BIC values signal a better fit), indicated that the common pathway model fit better than a correlated factors model and an independent pathway model (see Table 5 for the leaving latency and for saccade amplitude).

**Table 5:** Model fitting results for the leaving latency and saccade amplitude

Model	Leaving latency	Saccade amplitude
	BIC	BIC
Correlated factors	11152.858	11054.498
Common pathway	11137.967	11039.023
Independent pathway	11153.048	11056.071

Bayesian Information Criteria (BIC statistic)

In the common pathway model (for an example see Fig. 1), variance components (e.g. A, C, E) load on to a latent phenotypic factor that accounts for the observed and shared variance among the variables and where the residual variance (not explained by this latent factor) is also decomposed into genetic and environmental influences. In this case, it means that in the Gap-Overlap task, the covariance between the three conditions was best explained by one shared factor for all dependent variables (See Table 6 for shared and unique influences on all variables). This latent factor explained a substantial amount of the variance in all three variables of the leaving latency (gap = 0.71; baseline = 0.78; overlap = .81) and was primarily influenced by genetics (A = 0.66; 95% CI 0.52, 0.80) and moderately by non-shared environmental effects (E = 0.34; 95% CI 0.20, 0.48). There was no evidence for shared environmental effects on this variable. Unique residual variance to each condition was mostly explained by non-shared environment in all conditions (see Table 6), and only the Gap showing influence of genetic effects (A = 0.21; 95% CI 0.11, 0.31). None of the residual variances was explained by shared environment in any of the conditions. Notably, no unique genetic contribution was associated with the Overlap condition (Table 6), speaking against the hypothesis that this specific measure reflects a useful endophenotype for autistic traits (Sacrey et al. 2014).

**Table 6:** Common and unique parameter estimates of genetic and environmental to the common underlying factor and the unique measured variance in leaving latency and saccade amplitude according to the fitted Common Pathway model

	Common variance			Unique variance			
	*h*^2^	c^2^	e^2^	*h*^2^		c^2^	e^2^
Leaving latency							
Shared	0.66 (0.52, 0.80)*	0	0.34(0.20, 0.48)*	–		–	–
RG	–	–	–	0.21(0.11, 0.31)*		0	0.29 (0.21, 0.37)
RB	–	–	–	0.09(− 0.01, 0.19)		0	0.29 (0.19, 0.39)*
RO	–	–	–	0		0	0.33 (0.25, 0.41)*
Saccade amplitude							
Shared	0.82 (0.72, 0.92)*	0	0.18(0.08, 0.28)*	–		–	–
RG	–	–	–	0.07(− 0.00, 0.15)		0	0.23 (0.15, 0.31)*
RB	–	–	–	0.09(− 0.00, 0.19)		0	0.43 (0.31, 0.55)*
RO	–	–	–	0		0	0.37 (0.29, 0.45)*

*RG* Residual gap, *RB* residual baseline, *RO* residual overlap, *Shared* shared covariance, *h*^2^ additive genetics, *c*^2^ shared environment, *e*^2^ non-shared environment. Confidence intervals estimates at 95% are given in parentheses. *p < .05

For saccade amplitude (Table 6), the common latent factor for shared variance between conditions explained a substantial amount of the variance in each condition (gap = 0.82, baseline = 0.69, overlap = 0.78) and was highly influenced by genes (A = 0.82; 95% CI 0.72, 0.92). There were low contributions by non-shared environment (E = 0.18; 95% CI 0.08, 0.28) and there was no evidence for contributions by the shared environment. Residual variance unique to each condition was influenced only by non-shared environmental effects, ranging from low to moderate contributions (see Table 6).

### Phenotypic Correlations

In order to test the association between eye tracking variables (leaving latency and saccade amplitude) and the SRS total score for autistic traits (as well as for potential confounder variables age and IQ total score), Pearson correlations were computed using the sandwich estimator to estimate unbiased standard errors (given that observations were nested within twin pairs). For saccade amplitude, an average measure of all conditions was used (median), as we had no hypothesis for the separate conditions for this measure (see Table 7 for all correlations).

**Table 7:** Phenotypic correlations between the dependent variables and autistic traits, age and IQ

		SRS	Age	IQ
Leaving latency	Gap	− 0.01 (− 0.07, 0.05)	− 0.05 (− 0.16, 0.05)	0.07 (0.02, 0.12)*
	Baseline	− 0.03 (− 0.03, 0.09)	− 0.11 (− 0.21, − 0.01)*	0.03 (− 0.02, 0.08)
	Overlap	− 0.03 (− 0.09, 0.04)	− 0.04 (− 0.14, 0.06)	0.05 (− 0, 0.11)
Saccade amplitude average (all conditions)		− 0.00 (− 0.05, 0.05)	0.09 (− 0.01, 0.20)	0.07 (0.03, 0.12)*

*SRS* Social Responsiveness Scale. 95% confidence intervals (CI) estimates are given in parentheses. *p < .05

#### Autistic Traits (SRS)

Correlations between saccadic reaction times (leaving latency) in the conditions of the task and SRS scores were statistically non-significant (see Table 7; Fig. 2).

#### Age

Correlations between age and saccadic reaction times on the task (see Table 7) were significant only for the Baseline in the leaving latency, at a 95% confidence. Correlations were negative for all conditions, meaning that older participants had shorter leaving latencies. Correlations between saccade amplitude and age were also statistically non-significant.

#### IQ

Correlations with measured IQ were mostly non-significant (see Table 7), only the leaving latency in the Gap condition and saccade amplitude were significant but weak. It is notable, however, that the pattern suggests that higher IQ, if anything, is related to moving gaze later from the central target (descriptively, all conditions showed this pattern), and producing saccades that brings gaze closer to the peripheral target.

## Discussion

Being able to flexibly move ones gaze from one stimulus to another is a basic attentional skill that is important in the development of later attentional skills as arousal regulation and joint attention. If impaired, it has been postulated to create a knock-on effect of negative consequences across development by affecting the attentional skills that build on it and are part of more complex everyday social interactions (Keehn et al. 2013). The aim of the present study was to map the relative contribution of genes (the heritability) and environment to individual differences in visual disengagement and to evaluate the hypothesis that it is an endophenotype for autistic traits in the general population. Our hypotheses were that visual disengagement, as captured by the overlap condition in the Gap-Overlap task, would (1) have genetic influences and (2) would correlate positively with autistic traits (e.g. slower latencies = higher autistic traits). If visual disengagement is heritable and associated with autistic traits, it fulfills two of the criteria for an endophenotype (Gottesman and Gould 2003)—in this case for autism. The results only partially confirm the first of our hypotheses and contradict the second. First, the results showed that most of the covariance of the leaving latencies between the three conditions (Gap, Baseline, and Overlap) could be explained by a single common latent factor, which was primarily influenced by genetics. Further, as expected, we found unique genetic contributions to the individual conditions. Specifically, we found unique genetic contributions to the Gap condition, which is in line with the view that this condition uniquely captures alerting of attention. However, we found no unique effects for the Overlap condition, i.e. the condition supposedly capturing visual disengagement and which has been linked to autism in previous work. Second, we found no association with autistic traits. Thus, our data, which was based on a large sample of children, did not support the hypothesis that problems with visual disengagement is an endophenotype for social communication traits in the typical population (nor at its extreme: autistic traits).

Most of the covariance in leaving latencies across the conditions was explained by a common latent factor. This factor most likely reflects individual differences in oculomotor and attentional processes that are not sensitive to the experimental variations that characterize each condition. These are likely to include brain structures and networks involved in preparing and executing saccadic eye movements (e.g. pre-saccadic spike potential, pre-saccadic positivity; the frontal eye fields, supplementary eye fields, brainstem reticular formation, superior colliculus, cerebellum and other common areas), fixating, and even some aspect of visual disengagement for a discussion see (Csibra et al. 1997; Gómez et al. 1996; Munoz 2002; Dorris et al. 1997; Moster and Goldberg 1990). Although there is support for the independence of the alerting and orienting attention systems from Posner’s triadic network model (Fan et al. 2002, 2005), there is also evidence for interactions between said networks, where modification in one of the networks influenced performance on the others (Callejas et al. 2004, 2005; Fan et al. 2009). It is possible that the common latent factor might also be capturing some of this interaction.

The unique genetic contribution to the Gap condition most likely reflects individual differences in the “Gap effect”—a decrease in saccadic reaction times as a result of the introduction of a short temporal pause or gap between an already foveated stimulus and the subsequent peripheral stimulus (Tinsley and Everling 2002). Several explanations for the Gap effect have been proposed and a full discussion is beyond the scope of this paper. One prominent view holds that the temporal Gap serves as an alerting cue (Kingstone and Klein 1993; Posner and Petersen 1990; Csibra et al. 1997) and as a facilitator for saccade execution due to a predisengagement of attention. According to Posner’s model of attention, achieving and maintaining an alert state is supported by the alerting network (Posner and Petersen 1990), a function tightly linked to the norepinephrine system. Evidence from EEG studies in humans indicates specific ERP components represent likely neural correlates of the Gap effect, both of its unique “warning/readiness component” and of the likely “pre-disengagement component” (P1 response to stimulus off-set) (Gómez et al. 1996; Csibra et al. 1997). A likely neural correlate for this pre-disengagement effect is the peak decrease in discharge activity of fixation neurons in the superior colliculus displayed at 200–300 ms into the gap period. Before that period (< 100 ms and shorter gaps), the inhibitory activity of these neurons on the saccade-executing system is still high, and afterwards activity increases again after ~ 300 ms (Dorris and Munoz 1995).

The similar average performance in the Baseline and the Overlap conditions (Fig. 2), and the lack of unique genetic effects on the Overlap condition do not support the notion of this condition capturing a separate disengagement component—at least not one that is driven by genetic factors. This result, coupled with the results for the Gap condition, suggest that if one operationalizes visual disengagement as the contrast between Gap and Overlap [a frequently used approach, e.g. Elsabbagh et al. (2013)], the resulting difference score may not reflect genetic factors related to visual disengagement, but rather, genetic factors related to “the Gap effect” (e.g. alerting, responsivity to warning cues). In general, our results suggest that, despite its ostensive simplicity, the Gap-Overlap task is rather complex, both phenotypically and in terms of the pattern of contributing etiological factors, thus caution should be made when using difference scores between the conditions to obtain “pure” measures of specific cognitive constructs. Furthermore, the distribution for the Overlap condition appears multimodal. The latter has been well documented previously (Tinsley and Everling 2002; Fischer et al. 1993), and suggests that this condition reflects heterogeneous processes and not a unitary construct. Descriptively, it appears as the condition may have three peaks, two corresponding to the dominant peaks in the Gap and Baseline conditions, plus a third later peak not seen in the other conditions (Fig. 2). Our result also points to the danger of using arriving latencies as the primary dependent measure in this context, as it is likely to reflect not only visual disengagement, but also saccade accuracy, both of which seem to differ between the conditions.

Our results show that saccade amplitude is highly heritable, with no effect of the shared environment. This result is not consistent with the low heritability for saccade amplitude reported by (Katsanis et al. 2000), who found a significant contribution by shared environmental factors. It is unlikely that our study lacked power to detect these influences, as our sample size was four times larger than the one in the previous study (64 MZ, 48 DZ). We found no unique genetic or shared environmental effects related to individual conditions for this measure. Thus, while shorter amplitude was found for the Gap condition, the relative genetic and environmental influences for this variability appear to correlate with the ones responsible for overall variability in saccade amplitude across all conditions. We also observed an expected association between short latency and short amplitude. This is relatively common, especially in children of this age (Fischer et al. 1993; Klein and Foerster 2001; Posner and Petersen 1990), and can be seen as two sides of an unspecific alerting effect (fast but spatially imprecise responses).

Although several studies have linked slowed visual disengagement to autism (Courchesne et al. 1994; Elsabbagh et al. 2013)—for a review see (Sacrey et al. 2014)—others have not found support for this association (Fischer et al. 2014, 2016), including a recent meta-analysis (Johnson et al. 2016). Despite our large sample, we found no indication that problems with disengagement represent and endophenotype for typical variability in social communication nor at the extreme: high autistic traits in in the general population. This does not exclude the possibility that this measure is an endophenotype for autism at a younger age, which is suggested by some research on infants at risk (Elison et al. 2013; Elsabbagh et al. 2013). In the study by Elsabbagh et al. (2013), delayed visual disengagement was observed on those infants who later received a diagnosis of autism. Furthermore, disengagement was stagnant rather than decreasing with age, as would be expected in typically developing infants (Gredebäck et al. 2006) and older children (Munoz et al. 1998; Fischer et al. 1997), and which we also observed in our sample.

Taken together, by mapping the relative genetic and environmental contributions to individual differences or “architecture” of a basic attentional construct, this study represents a new conceptual approach within the field of cognitive neuroscience. Most of the variance across the conditions was attributable to one common and predominantly genetic factor. This factor most likely captures basic oculomotor and attentional processes common to all conditions. Only the Gap condition showed unique genetic influences, arguably reflecting unique heritability of alerting due to the warning signal component this condition presents. Our results do not support the link between slower visual disengagement and typical variability in social communication in late childhood and early adolescence. A critical next step involves assessing this task in infant twins to establish whether problems with attention are heritable in infancy, and if they predict the later emergence of socio-communicative problems and other traits linked to autism.

## Electronic supplementary material

Below is the link to the electronic supplementary material.

Supplementary material 1 (DOCX 4906 KB)
