# Supplementary material for: Visual Disengagement: Genetic Architecture and Relation to Autistic Traits in the General Population
Source: J Autism Dev Disord. 2019 Mar 11;50(6):2188–200. doi: 10.1007/s10803-019-03974-6 (PMC7261271; doi:10.1007/s10803-019-03974-6)
Supplement: Supplementary file 1 — Supplementary material 1 (DOCX 4906 KB) [file 10803_2019_3974_MOESM1_ESM.docx]

**Figure 1.** Maximum parental educational level attained in this study’s sample (n=492) compared to the CATSS sample. *CATSS sample mother (n=18982), father (n=17582)


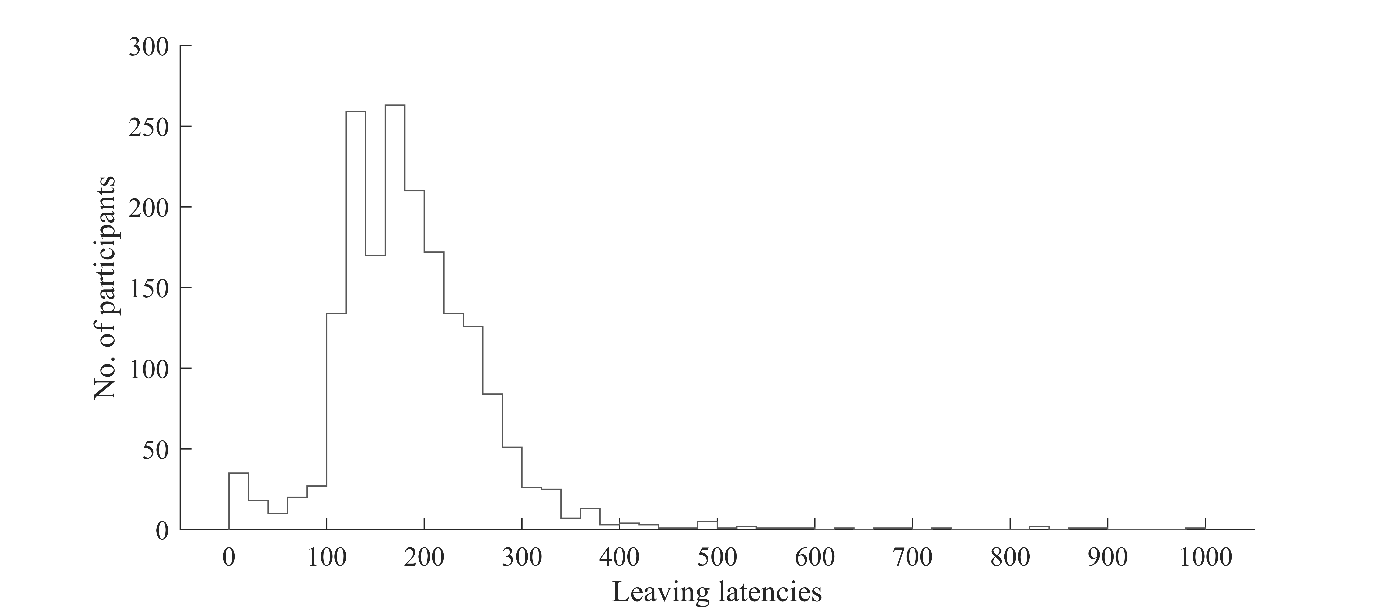


**Figure 2.** Histogram of individual mean saccadic reaction times of leaving latencies (all conditions)

**Table 1.** Correlations between leaving latencies across task conditions, and correlations between arriving latencies across task conditions

| Condition | Gap | Baseline | Overlap |
| --- | --- | --- | --- |
| Leaving latency |  |  |  |
| Gap | 1 |  |  |
| Baseline | .5 (.42 , .58) | 1 |  |
| Overlap | .55 (.47 , .62) | .62 (.56 , .68) | 1 |
| Arriving latency |  |  |  |
| Gap | 1 |  |  |
| Baseline | .47 (.39 , .55) | 1 |  |
| Overlap | .48 (.40 , .55) | .5 (.42 , .58) | 1 |

95% confidence intervals (CI) estimates are given in parentheses. **p* < .05

**Table 2.** Means and standard deviations (SD) of the leaving latency, arriving latency and saccade amplitude for the three conditions

|  | Gap | Baseline | Overlap |
| --- | --- | --- | --- |
| Leaving latency |  |  |  |
| Mean (SD) | 153.75(30.48) | 196.23(30.72) | 201.83(51.18) |
| Skewness | 1.108 | 0.271 | 0.240 |
| Kurtosis | 1.247 | 0.514 | -0.757 |
| Shapiro-Wilk | *W*(578)=0.917** | *W*(578)=0.982** | *W*(578)=0.972** |
| Arriving latency |  |  |  |
| Mean (SD) | 262.59 (45.32) | 259.82 (38.35) | 299.33 (44.81) |
| Skewness | 0.214 | 1.245 | 0.779 |
| Kurtosis | 0.565 | 3.626 | 3.817 |
| Shapiro-Wilk | *W*(578)=0.987** | *W*(578)=0.929** | *W*(578)=0.963** |
| Saccade amplitude |  |  |  |
| Mean (SD) | 11.13°(1.03°) | 11.79°(0.65°) | 11.38°(1.02°) |
| Skewness | -1.077 | -2.668 | -1.714 |
| Kurtosis | 1.175 | 11.871 | 3.036 |
| Shapiro-Wilk | *W*(578)=0.920** | *W*(578)=0.781** | *W*(578)=0.820** |

Leaving and arriving latency means and SD’s (in parentheses) are in milliseconds. Saccade amplitude is in visual degrees (°). Skewness and kurtosis are reported for all variables and conditions (for Saccade amplitude, these values are computed from the data prior to conversion to visual degrees). ***p*<.001.

For the Leaving latency (Figure 2), the ANOVA results showed that saccadic reaction times differed significantly between three conditions (*F*(1.71, 988.83)=584.76, *p<*.001; sphericity not assumed). Pairwise comparisons indicated that this was the case between all conditions and showed that saccadic reaction times (latencies) significantly differ in a stepwise manner, with latencies in the gap being significantly lower than those in the baseline (p<.001), and baseline latencies significantly lower than overlap latencies (p=.002). Gap latencies were also significantly lower than overlap latencies (p<.001).


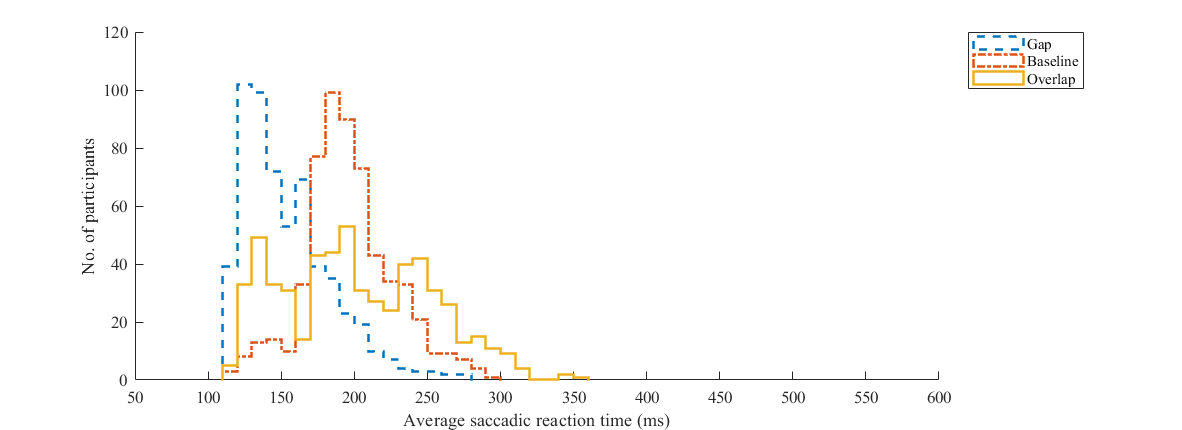


**Figure 3.** Histogram of individual mean saccadic reaction times of leaving latencies in the three conditions.

For the arriving latency (Figure 3), results showed a significant main effect of condition, suggesting a difference in saccadic reaction times between conditions (*F*(2, 1154)=313.107, *p*<.001). Pairwise comparisons indicate that Baseline and Gap conditions were not significantly different from each other (*p*=.359), while the saccadic reaction times (latencies) in both were significantly slower than those in the Overlap condition (*p<*.001, in both).


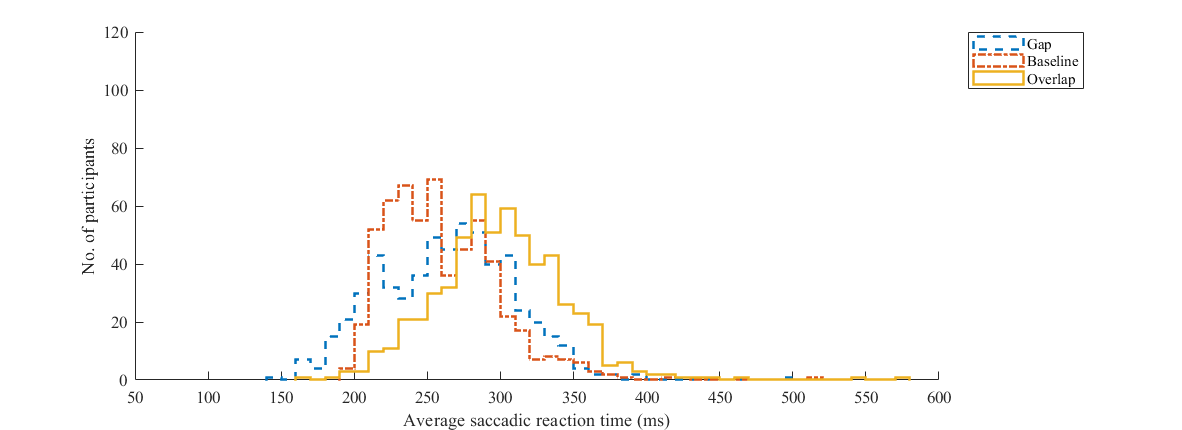


**Figure 4.** Histogram of individual mean saccadic reaction times of arriving latencies in the three conditions.

The repeated measures ANOVA results for Saccade amplitude (Figure 4) showed a main effect of condition, (*F*(2, 1154)=192.349, *p*<.001), suggesting that saccade amplitudes are significantly different between conditions. Pairwise comparisons showed that there were significant differences between all conditions (Gap<Overlap<Baseline), with shorter saccadic amplitudes in the Gap condition (p<.001), a larger average for those in Baseline (p<.001), and amplitudes in the Overlap condition falling somewhere in the middle yet significantly different from those in the other two conditions (p<.001 for both).


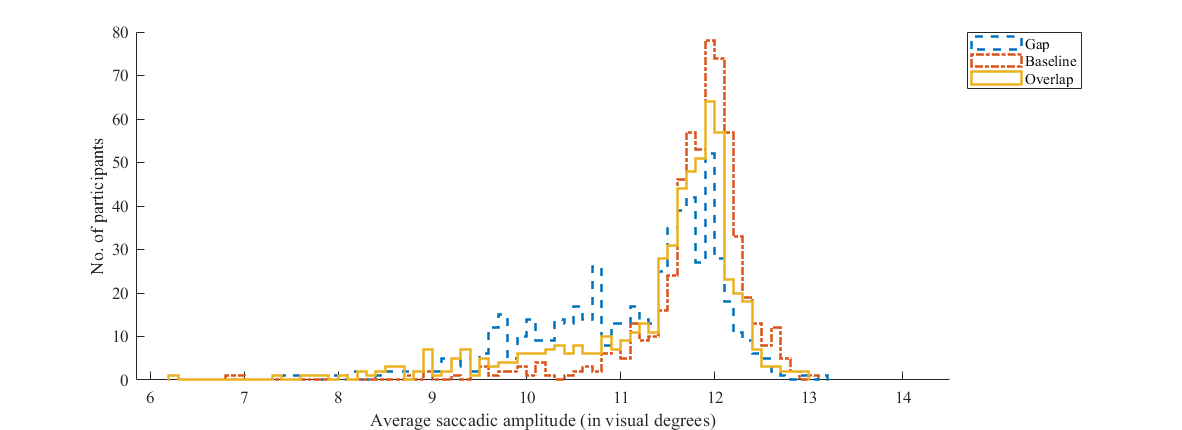


**Figure 5.** Histogram of individual mean saccadic amplitudes in the three conditions.


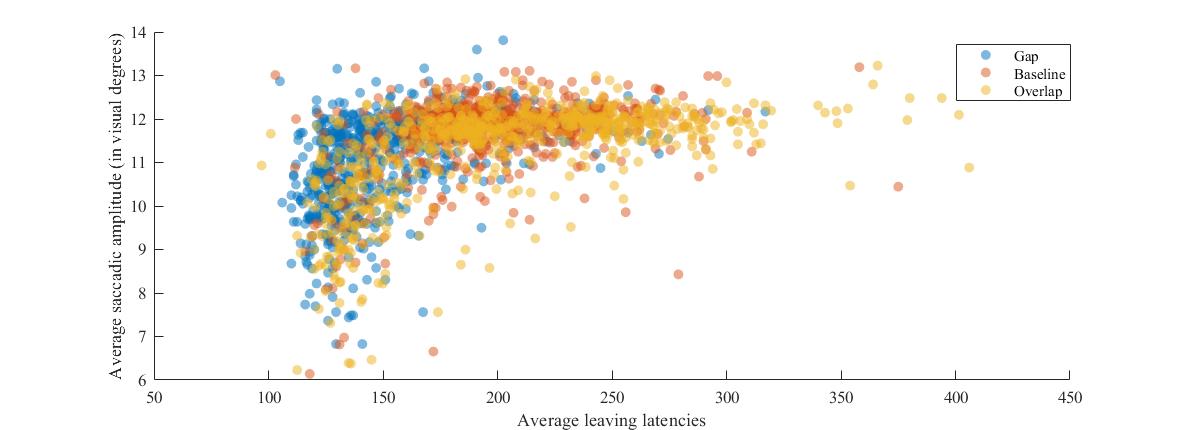


**Figure 6.** In the figure, individual averages (mean leaving latencies in milliseconds) along the X axis and saccadic amplitudes in pixels along the Y axis.
